# Metagenomics datasets of water and sediments from eutrophication-impacted artificial lakes in South Africa

**DOI:** 10.1038/s41597-024-03286-0

**Published:** 2024-05-06

**Authors:** Grace Nkechinyere Ijoma, Henry Joseph Oduor Ogola, Preenan Pillay, Kalonji Abondance Tshisekedi, Memory Tekere

**Affiliations:** 1 Department of Environmental Science, College of Agricultural and Environmental Sciences, University of South Africa, Roodepoort, Gauteng, South Africa; 2 School of Agriculture and Sciences, Jaramogi Oginga Odinga University of Science and Technology, Bondo, Kenya; 3 School of Molecular and Cell Biology, Faculty of Science, University of Witwatersrand, Johannesburg, Gauteng, South Africa

**Keywords:** Biodiversity, Environmental biotechnology

## Abstract

We present metagenomes of 16 samples of water and sediment from two lakes, collected from eutrophic and non-eutrophic areas, including pooled samples enriched with phosphate and nitrate. Additionally, we assembled 167 bacterial metagenome-assembled genomes (MAGs). These MAGs were de-replicated into 83 unique genomes representing different species found in the lakes. All the MAGs exhibited >70% completeness and <10% contamination, with 79 MAGs being classified as ‘nearly complete’ (completeness >90%), while 54 falling within 80–90% range and 34 between 75–80% complete. The most abundant MAGs identified across all samples were Proteobacteria (n = 80), Firmicutes_A (n = 35), Firmicutes (n = 13), and Bacteriodota (n = 22). Other groups included Desulfobacteria_I (n = 2), Verrucomicrobiota (n = 4), Campylobacterota (n = 4) and Actinobacteriota (n = 6). Importantly, phylogenomic analysis identified that approximately 50.3% of the MAGs could not be classified to known species, suggesting the presence of potentially new and unknown bacteria in these lakes, warranting further in-depth investigation. This study provides valuable new dataset on the diverse and often unique microbial communities living in polluted lakes, useful in developing effective strategies to manage pollution.

## Background & Summary

South Africa has a significant number of artificial lakes primarily designed to manage water runoff from urban areas to prevent flooding. Many of these lakes, located in different areas, serve as recreational spaces for residents. Since the Gauteng province has the South Africa’s largest population^1^, direct and indirect interactions with these water bodies are extensive^2^. Internationally, lake ecosystems are known for their susceptibility to rapid and predictable changes in their microbiome structure and diversity, primarily due to their sensitivity to perturbations associated with urbanization-induced human inputs^3,4^. For example, lakes have been identified as reservoirs of pathogenic bacteria and ecologically harmful bacterial communities, often linked to eutrophication^4^. With the high population density in Gauteng, the runoff water from urban areas into these lakes poses a serious risk to the environmental ecology and human health. Therefore, gaining comprehensive insight into the diversity and distribution patterns of microbial communities within the largest lakes in Gauteng is crucial.

The emergence of metagenomics has brought a paradigm shift in the study of microbial communities within complex ecosystems. This powerful genetic approach allows for the discovery of unknown taxa, thereby enriching knowledge on diverse functional community molecular content across environmental gradients and compartments without the need for microbial culturing^5^. Shotgun metagenomic sequencing, in particular, offers several advantages over targeted amplicon analysis based on 16S rDNA or ITS gene, including enhanced detection of bacterial and fungal species, increased detection of diversity, and increased prediction of genes^6^. In recent years, various assembly and binning tools have been developed^7^, enabling a transition in microbiome studies from gene-centric approaches to genome-resolved metagenomics. This evolution has given rise to population-level genomics through metagenome-assembled genomes (MAGs)^8–10^. This strategy allows for adequate read coverage that enables the assembly of short sequence reads into contigs, which are then binned into MAGs, facilitating the reconstruction of genomes for both well-established species and uncultured taxa^11^. This expands our understanding of microbial phylogeny and metabolic diversity. This method has been extensively employed to identify a large number of uncultured microbial communities from complex environmental samples^12,13^, including lakes affected by human activities^3,9,14^.

Unfortunately, our thorough literature search has revealed an apparent absence of documented metagenomic studies dedicated to South African lakes. A detailed and high-quality metagenomic record of such lake microbiomes will be pivotal in establishing a baseline for evaluating alterations and anthropogenic influences on water quality, providing crucial insights for the effective management of these vital water bodies. Moreover, such data can aid in the identification of microbial health hazards, and may serve as a foundation for future monitoring, utilizing the *in-situ* microbiomes as indicators of environmental health.

**Fig. 1:**
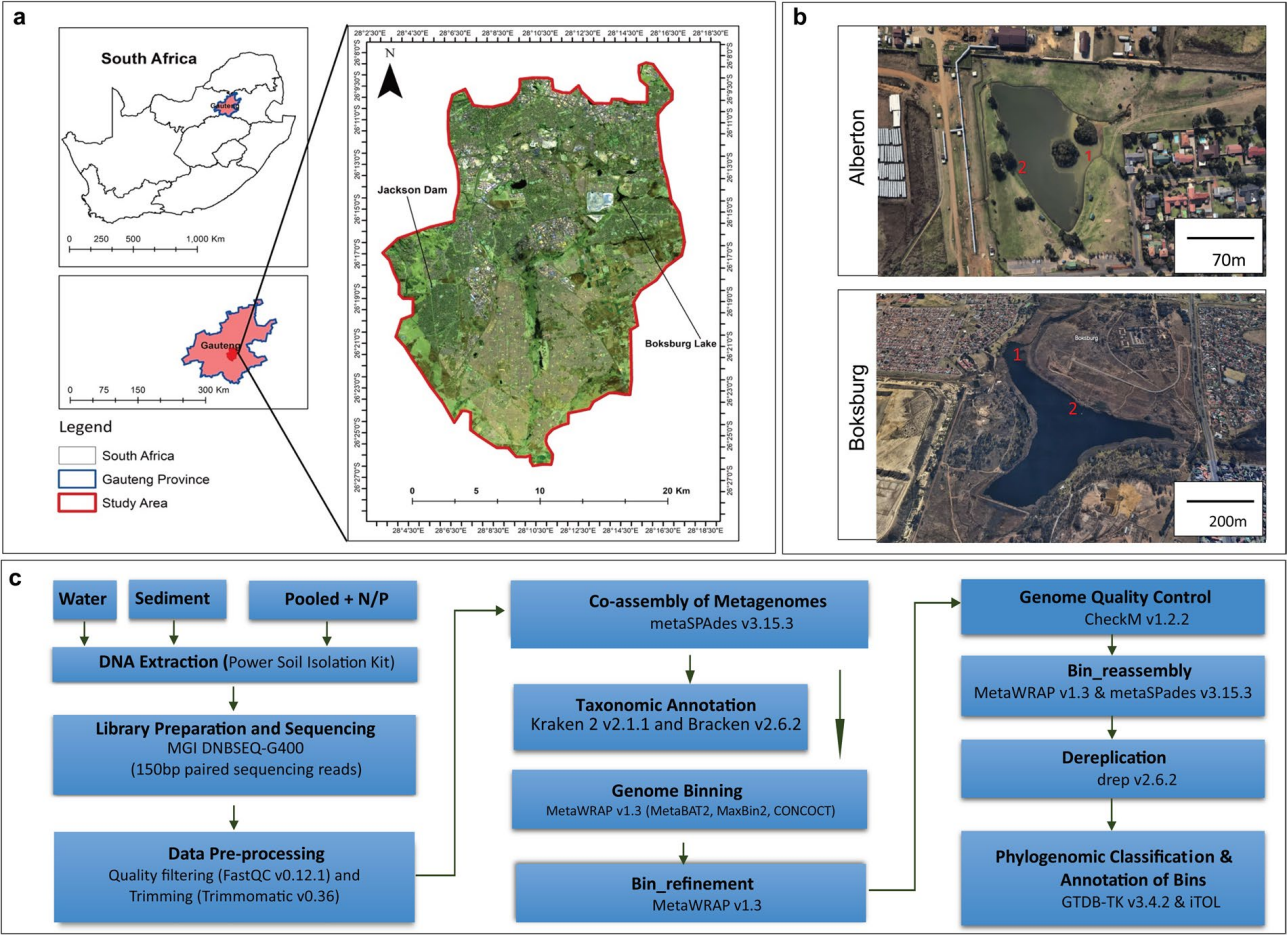
Sample Collection and Metagenomic Analysis. (**a**) Geographical location of the sample sites. (**b**) The sampling area within each lake. (**c**) Schematic representation for the metagenomic analysis conducted.

**Fig. 2:**
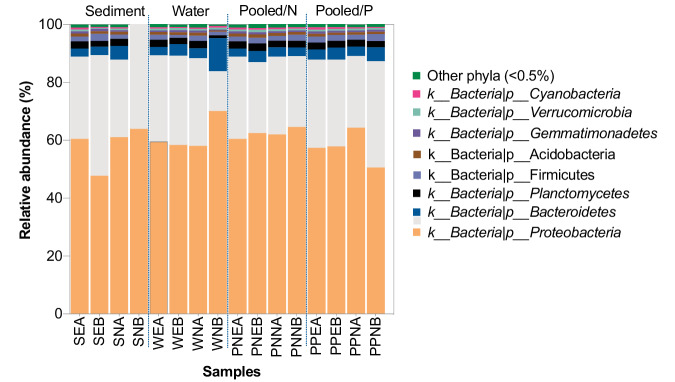
Relative abundance of bacterial phyla in the lake sediment and water samples based on Kraken2/ Bracken classification.

Here, we present 58.6 Gb (average 3.7 Gb) shotgun metagenome datasets of 16 water and sediment samples from Boksburg and Alberton Lakes (Fig. 1a,b), including eutrophic and non-eutrophic areas, as well as pooled samples enriched with phosphate and nitrate (Table 1). The sample information, sequencing quality metrics, and assembly statistics of the shotgun metagenomic data are shown in Table 1. Taxonomic annotation with Kracken2/Bracken^15^ identified 94.0%, 4.6% 1.37, and 0.026% of the classified sequences across all samples as Bacteria, Archaeal, Fungal, and Viral taxa, respectively. Among the bacterial sequences, 39 phyla were identified, with Proteobacteria (59.3%), Actinobacteria (28.8%), Bacteroidetes (4.2%), Planctomycetes (2.2%), and Firmicutes (1.8%) being the most abundant (>1% relative abundance). The relative abundance of bacterial phyla across the samples is provided in Fig. 2.

**Table 1:** Accession numbers, sequencing information, and assembly statistics of shotgun metagenomes of sediment and water samples from Lake Alberton and Boksburg in Gauteng Province, South Africa.

Sample	Source/ Enrichment	Pollution state	Quality reads (Millions)	Bases (G)	Size (Gbp)	NCBI SRA accession
**Alberton**						
SEA	Sediment	Eutrophic	16.251	4.9	2.8	SRX23132301
SNA	Sediment	Non-eutrophic	13.416	4	2.1	SRX23132302
PNNA	Pooled/Nitrate	Non-eutrophic	26.081	7.8	4.3	SRX23132309
PPEA	Pooled/Phosphate	Eutrophic	21.163	6.3	3.4	SRX23132310
PPNA	Pooled/Phosphate	Non-eutrophic	24.427	7.3	3.9	SRX23132311
WNA	Water	Non-eutrophic	25.022	7.5	3.9	SRX23132304
WEA	Water	Eutrophic	19.864	6	3.1	SRX23132305
PNEA	Pooled	Eutrophic	20.24	6.1	3.3	SRX23132308
**Boksburg**						
PNEB	Pooled/Nitrate	Eutrophic	29.586	8.9	4.8	SRX23132312
PNNB	Pooled/Nitrate	Non-eutrophic	25.784	7.7	4.1	SRX23132313
PPEB	Pooled/Phosphate	Eutrophic	22.295	6.7	3.5	SRX23132314
PPNB	Pooled/Phosphate	Non-eutrophic	24.472	7.3	3.8	SRX23132315
SEB	Sediment	Eutrophic	20.681	6.2	3.3	S RX23132316
SNB	Sediment	Non-eutrophic	20.717	6.2	3.3	SRX23132303
WNB	Water	Non-eutrophic	27.06	8.1	4.4	SRX23132306
WEB	Water	Eutrophic	28.146	8.4	4.6	SRX23132307

The average Phred score and read length for all samples was 36 and 150 bp, respectively.

**Fig. 3:**
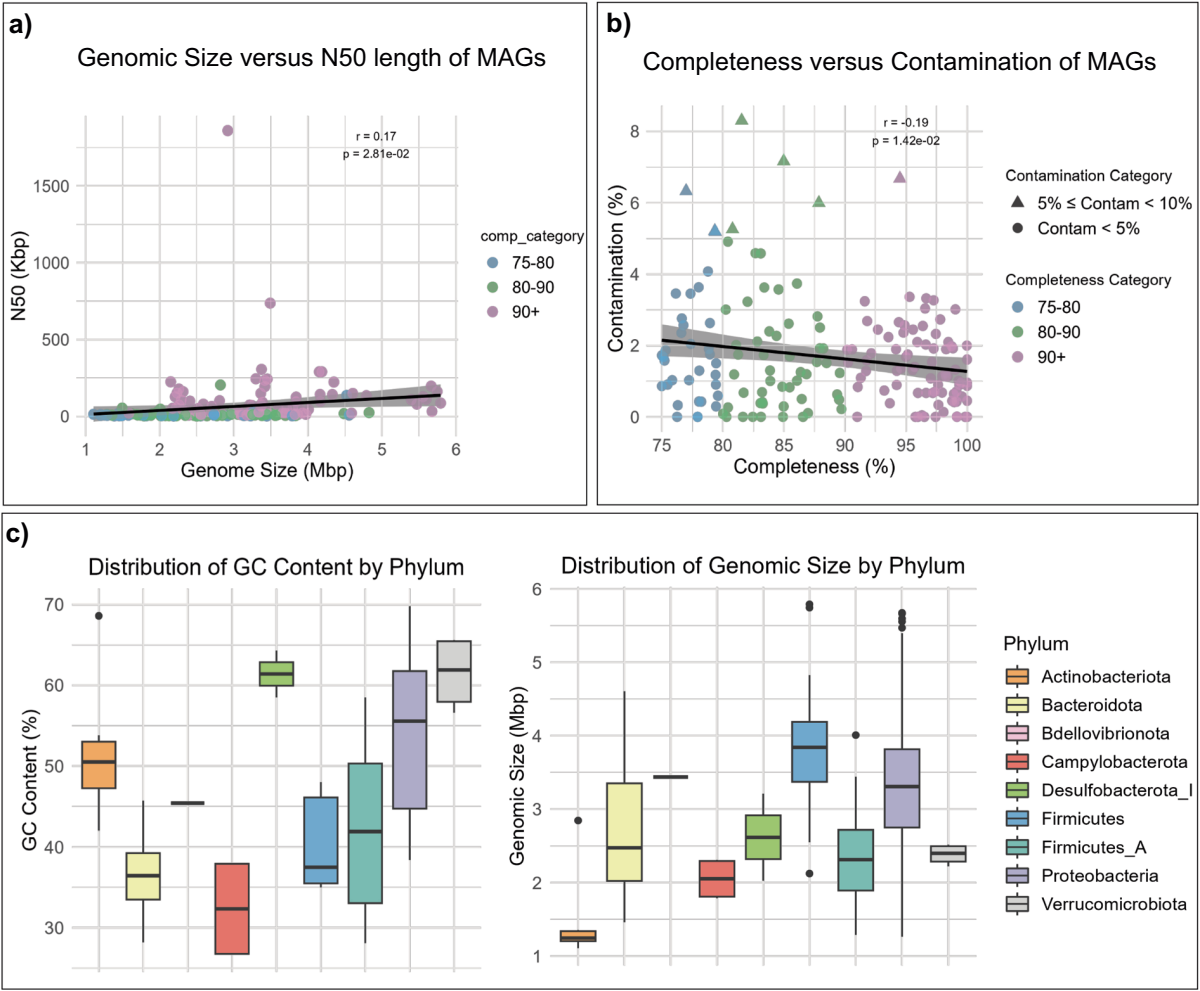
Overview of the MAGS. (**a**) The relationship between genomic size and N50 length among MAGs. (**b**) The relationship between the completeness and contamination of MAGs. (**c**) Boxplots compare the distribution of genomic size and GC content among MAGs at the phylum level.

Using metaSPAdes v3.15.3 pipeline^16^, we reconstructed 167 metagenome-assembled genomes (MAGs) from the shotgun metagenomes generated in this study. The assembly quality metrics of the MAGs is summarised in Supplementary Information Table S1. All the MAGs had completeness of >75% with a contamination <10%), meeting the medium quality of the minimum information about a metagenome-assembled genome (MIMAG) standard^17^. Within the MAGs, 80 (48%) were near complete (completeness >90%), 54 (32%) were between 80%–90% completeness, and 34 (20%) were between 75%–80% completeness. Notably, 159 (95%) MAGs had <5% contamination, and 8 (5%) MAGs showed no contamination. The assembly quality was also high, as a total of 109 MAGs (64%) had an N50 length of greater than 10,000 bp, with the longest value reaching 1.85 Mbp (Supplementary Information Table S2). The genome size of the MAGs ranged from 1.10 to 5.79 Mbp, with an average value of 2.94 Mbp (Supplementary Information Table S2). Overall, Verrucomicrobiota had the highest GC content (average 69.8%), while Campylobacterota had the lowest GC content (26.7%) (Fig. 3c, Supplementary Information Table S2). However, no significant correlation was observed between genome size and N50 length, as well as the completeness and contamination (Fig. 3a,b).

**Fig. 4:**
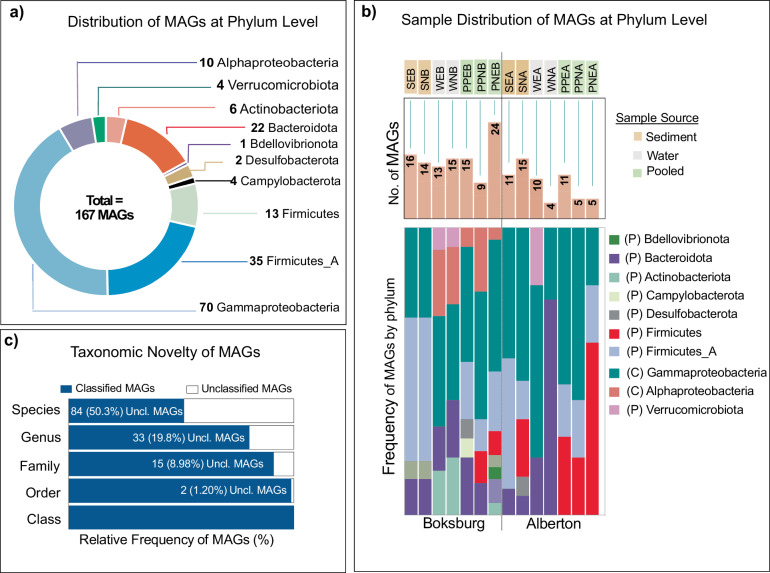
Taxonomic annotation and novelty of MAGs. (**a**) Phylogenomics-based taxonomic classification of the 167 MAGs dataset at the phylum level. The phylum Proteobacteria have been split at class level. (**b**) Stacked bar plot of the relative distribution of MAGs at phylum level across different samples. The top bars represent the number of MAGs in each sample. (**c**) Stacked bar plot for novelty quantification of 167 MAGs at different taxonomic ranks.

**Fig. 5:**
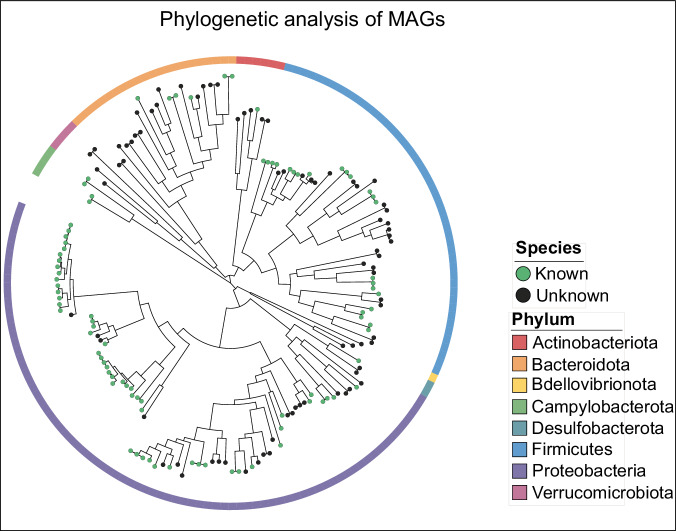
Phylogenetic tree of bacteria at species-level MAGs (n = 167). The colors within the circle at the ends of the phylogenetic branches represent known (green) and unknown (black) species. The outer ring represents the various phyla of the MAGs as per the labels in the legend.

In total, all the MAGs were phylogenomically classified into 167 bacteria based on the Genome Taxonomy Database (GTDB-Tk)^18^ (Figs. 4, 5; Supplementary Information Table S2). Nine phyla were identified, with the most abundant being Proteobacteria, including class Gammaproteobacteria, (n = 70) and Alphaproteobacteria (n = 10), Firmicutes_A (n = 35), and Bacteriodota (n = 22) (Fig. 4a). The distribution of the MAGS at phylum in the different samples of the two lakes is illustrated in Fig. 4b and Supplementary Information Table S2. Examining sampling locations, the microbial community of Alberton samples was predominantly characterized by the Proteobacteria phylum, accounting for a significant 50.79% of the identified bacteria. Other abundant taxa included the Firmicutes_A and Firmicutes phyla at 17.46% and 15.87% relative abundance, respectively. Bacteroidota also had a notable presence, capturing 11.11% of the community. A similar trend, with subtle variations in microbial landscape influenced by sample type, enrichment and eutrophic state, was observed in Boksburg (Fig. 4b). Proteobacteria remained dominant, representing 47.17% of the total MAGs. However, Firmicutes_A occupied a larger proportion compared to Alberton, making up 22.64% of the microbial diversity. The representation of Bacteroidota was consistent with Alberton, constituting a significant 14.15% (Fig. 4b; Supplementary Information Table S2).

For pooled samples, Proteobacteria also emerged as the predominant phylum, constituting 50.72% of the total. Firmicutes_A and Firmicutes followed representing 18.84% and 14.49% respectively. Bacteroidota was also an abundant taxon, accounting for 8.69%. In the sediment samples, Proteobacteria occupied 40.35%, but the Firmicutes_A phylum took precedence as the dominant taxa, representing 38.60% of the microbial content (Supplementary Information Table 2). Bacteroidota was also substantial, making up 10.53%. In contrast, water column samples were dominated by Proteobacteria at 55.81% relative abundance, followed closely Bacteroidota at 23.26%. Other significant taxa included Actinobacteriota and Verrucomicrobiota, which constituted 11.63% and 9.30% of the microbial population, respectively. Interestingly, a significant number of MAGs could not be classified at order (n = 2; 1.2%), family (n = 15; 8.98%), genus (n = 33; 19.8%) and species level (n = 82; 48.5%), suggesting that the majority of these MAGs could represent novel taxa and should be investigated further (Figs. 4c, 5).

The presented study has uncovered a diverse array of novel microbial genomes within artificial lakes in South Africa, identified in both water and sediment samples. Furthermore, our datasets will be used to generate novel hypotheses regarding the connections between lake microbiomes and human activities in the watershed. Thereby, creating an understanding of the microbial species diversity, structure, and function, within these South African lakes, which can be leveraged for the implementation of advanced monitoring and environmental management strategies.

## Methods

### Sampling sites and sample collection

In 2022, during the month of February (Summer), we collected water column and sediment samples from two urbanization-impacted artificial lakes (Boksburg, 26° 13′15.24″ S, 28°14′51.36″ E & Alberton, 26°18′30.8″ S, 28°05″30.0″ E) within Gauteng, South Africa (Fig. 1a,b). In each lake, runoff and central samples were identified based on topography (Fig. 1b) and collected from locations indicated in Fig. 1c (Boksburg (Runoff: 26° 14′00″ S, 28°14′14″ E, Central: 26° 14′10″ S, 28°14′21″ E), Alberton (Runoff: 26°18′28″ S, 28°05″31″ E, Central: 26°18′28″ S, 28°05″28″ E)). At each sampling site, triplicate samples of water (10–15 cm depth) and sediment samples (at the lake bottom) were colle were collected in sterile 5 L sodium bottles (Corning® Gosselin™, NY, USA) and plastic bags, placed on ice and immediately transported to the laboratory for processing to maintain DNA quality.

### Shotgun metagenomics and data analysis

#### Metagenomic sequencing

The DNA sample processing involved several key steps, starting with DNA extraction and purification, followed by library preparation and high-throughput shotgun sequencing. Initially, environmental DNA was purified from water and sediment samples using the DNeasy® PowerSoil Pro Kit (Qiagen, Germany) as per the manufacturer’s instructions. Subsequently, DNA libraries were prepared following the MGIEasy Universal DNA Library Prep Set User Manual v1 Protocol (MGI Tech Co., Shenzen, China). This involved fragmenting the genomic DNA using the Covaris M220 Focused-Ultrasonicator (Covaris, Brighton, UK), followed by end repair and A-tailing of the sheared DNA. Following this, adapters were ligated following the protocols outlined in the MGIEasy DNA Adapters kit, and the ligated DNA was purified using DNA Clean Beads provided in the kit. PCR amplification was then performed on the purified, adapter-ligated DNA, followed by a second round of purification using magnetic beads. The quality of the PCR products was assessed using the Qubit dsDNA HS Assay Kit (Thermo Fisher Scientific, Waltham, MD, USA). Subsequently, the PCR products underwent another round of purification, followed by denaturation and ligation to produce single-strand circular DNA libraries. Barcode libraries were combined in equal proportions to create DNA Nanoballs (DNB), which were sequenced using DNBSEQ-G400 sequencer technology (MGI Tech Co., Shenzen, China), following the manufacturer’s guidelines. This sequencing was carried out at the Biotechnology Platform, Agricultural Research Council in Pretoria, South Africa. The raw data can be accessed at the NCBI database under the Bioproject ID PRJNA1022586^19^ and Sequence Read Archive (SRA) accession number SRP482505^20^.

#### Quality control and assembly

Figure 1c illustrates the workflow for bioinformatic analysis of the generated NGS data. Raw sequence reads underwent a quality assessment utilizing FastQC v0.12.1^21^ (parameters: default) and MultiQC v1.15^22^ (parameters: default). Subsequent data processing involved the eliminating adapter sequences, human reads and the exclusion of sub-par quality reads. Specifically, Trimmomatic V0.36^23^ (parameters: default) was used to remove reads that fell short of 30 bp or exhibited an average quality score below 20. Initially, the unmapped high-quality reads were taxonomically classified using Kraken2 v2.1.1^15^ (parameters: default) with the standard database (which includes all bacterial, archaeal, and viral genomes from NCBI; accessed December 1, 2024). Abundances were re-estimated at the species level using Bracken v2.6.2^24^ using default parameters. The retained high-quality reads were also co-assembled using metaSPAdes v3.15.3^16^ (parameters: default). The resultant assembly’s integrity and standard were evaluated with QUAST v5.2.0^25^.

#### Binning of metagenomic data and its refinement

The process of metagenomic binning employed tetranucleotide frequencies, coverage, and GC content as criteria. This analysis was conducted using the MetaWRAP v1.3^26^ pipeline in its default mode, which include tools like MaxBin v2.0^27^, metaBAT2^28^, and CONCOCT v1.0.0^7^. To improve bin quality, the MetaWRAP-Bin_refinement module was applied with settings -c 70 and -x 10. These settings helped to filter out low-quality segments and potential contaminants. The completeness and possible contamination of the binned segments were then assessed using CheckM v1.2.2^29^, which is integrated into the MetaWRAP pipeline. Subsequently, the bins were reassembled using the MetaWRAP-reassemble_bins module with parameters -c 70 -x 10. This step further improved the quality and contiguity of the assembled genomes. Finally, the polished bins were dereplicated using dRep v2.6.2^30^ to ensure that only unique genomes were included in the final analysis. De-replication was performed based on a 95% average nucleotide identity (ANI) benchmark, resulting in a final set of 167 distinct MAGs.

#### Phylogenetic assessment and MAGs taxonomy

To assign taxonomic classifications to the MAGs, the *classify_wf* function of GTDB-Tk v3.4.2^18^ with the reference database GTDB release207 v2, all in their default settings. This tool utilizes 120 bacterial marker genes to construct a phylogenetic tree, which visually represented the evolutionary relationships between the 167 identified bacterial MAGs. For easier visualization and interpretation, the tree was annotated with iTOL v5^31^.

## Data Records

The raw shotgun sequencing datasets and the sequence data for 167 MAGs have been deposited in the National Center for Biotechnology Information (NCBI) database under the Bioproject ID PRJNA1022586^19^ and Sequence Read Archive (SRA) accession number SRP482505^20^. Additionally, the sequence data of 167 MAGs have been deposited in Figshare^32^.

## Technical Validation

Before analyzing the data, we conducted quality checks on the purified environmental DNA. We used a NanoDrop 2000 spectrophotometer to measure the DNA concentration and an A_260_:A_280_ ratio (which assesses the purity of the DNA). Only samples with a ratio between 1.8 and 2.0 and a concentration of 20–150 ng/μl were used for library preparation and sequencing. These libraries were then quantified using a Qubit 4 fluorometer and the Qubit™ dsDNA HS Assay Kit. Quality distribution showed Q30 aggregated percentage of bases to be higher than 89 for all metagenomes. PHRED score was 36 for all samples (Supplementary Table S1). CheckM^29^ v1.2.2 was used to assess the completeness and potential contamination of the draft genomes.

### Supplementary information

Table S2.

Table S1.

## Data Availability

Custom-designed scripts were not used to generate or process any data presented. The publicly available software was used in their default settings unless stated otherwise within the text.
